# Abelchia: inability to belch/burp—a new disorder? Retrograde cricopharyngeal dysfunction (RCPD)

**DOI:** 10.1007/s00405-021-06790-w

**Published:** 2021-04-24

**Authors:** Yakubu Karagama

**Affiliations:** HCA Shard London, BMI Blackheath, London & Alexandra Hospital, Manchester, Guy’s and St Thomas’ Hospitals NHS Trust, London, UK

**Keywords:** Inability to burp-belch, Abelchia, Retrograde cricopharyngeal dysfunction - RCPD, Botulinum toxin A injection, Botox

## Abstract

**Case series:**

This is retrospective case series involving 72 patients who presented with symptoms associated with inability to burp. The following symptoms was described by almost all the patients; retrosternal pain after eating or drinking, bloating feeling in the stomach, gurgling noise in the throat, excessive flatulence. These symptoms are worse with fizzy/carbonated drinks and beer. A full clinical history and examination plus endoscopic and in some cases barium a swallow radiological investigation was done.

**Procedure:**

The surgery was performed under a general anaesthesia for all cases. Suspension pharyngoscopy in supine position using a Weerda diverticuloscope to identify the cricopharyngeal bar muscle. High dose of botulinum toxin A (botox) 100 iu was injected into the cricopharynxgeus muscle under a general anaesthesia.

**Results:**

A total of 72 patients were diagnosed and undergone surgery between November 2016 and December 2020. There were 50 male and 22 female patients. Their average age was 30 (range 18–68 years old). All patients were able to burp again within first 4 weeks of the injection. This persisted even after the Botox worn off beyond the 3 months in 96% of cases. The average follow-up was 24 months post injection with longest follow-up 48 months (range 1–48 months).

**Conclusion:**

The author reported a new condition of inability to burp due to failure of the cricopharyngeal sphincter to relax spontaneously and outcome of treatment using botulinum toxin A injection into the cricopharyngeus muscle. It is expected that the paralysing action of botulinum toxin injection last approximately 3 months. However, this group of patients seem to be cured even after the effect of the botox is worn off. The author therefore postulated that there might me some neural dysfunction that inhibits the brain to send signals to the cricopharyngeal sphincter to initiate burping. Once burping is re-established with the help of botox injection, spontaneous burping seems to occur and sustained even after the botox is worn off.

## Introduction

Patients with inability to burp or abelchia present typically with symptoms of bloating, abdominal and retrosternal or chest pain/discomfort, gurgling noise in the throat, excessive flatulence [1, 2]. The basic examination should include a flexible nasal pharyngosocpy and transnasal oesophagoscopy which may show oesophageal gas distention. Laboratory investigations for instance PH measurement or manometer or radiological examination like barium swallow may show changes [3] but the absence of abnormality does not rule out this condition because there may not have been gas trapped at the time of investigation as this is a functional condition. Inability to burp is a dysfunction of the cricopharyngeal muscle failing to recognise and release the trapped gas below upper oesophageal sphincter leading to retrograde dysfunction of the cricopharyngeal muscle. The cricopharyngeal muscle is an elastic-like muscle fibres which forms the circular upper oesophageal sphincter. This acts like a valve to oesophageal inlet. The cricopharyngeal sphincter is usually in a state of contraction and only relaxing to allow passage of food down or during burping/belching. The belching/burping reflex requires relaxation of the upper oesophageal sphincter [3]. In people with inability to burp, the cricopharyngeal muscle fails to relax during burping; therefore, gas get trapped in the oesophagus and progressively into the stomach and bowels.

## Case series report

This is a retrospective case series involving patients who presented with symptoms associated with inability to burp. This work was conducted in three separate institutions by the same author. The following symptoms were described by almost all the patients; Retrosternal/chest pain after eating or drinking, bloating feeling in the stomach, gurgling noise in the throat, excessive flatulence. These symptoms are worse with fizzy/carbonated drinks and beer. Some patients have forced themselves to burp by sticking their finger into the back of their throat. Others get temporary relief by lying supine on their left side for about an hour to allow the gas to pass down the alimentary tract, which later gets expelled as excessive flatulence. The onset of symptoms in most patients was from 2 to 8 years and since birth in some patients.

**Fig. 1:**
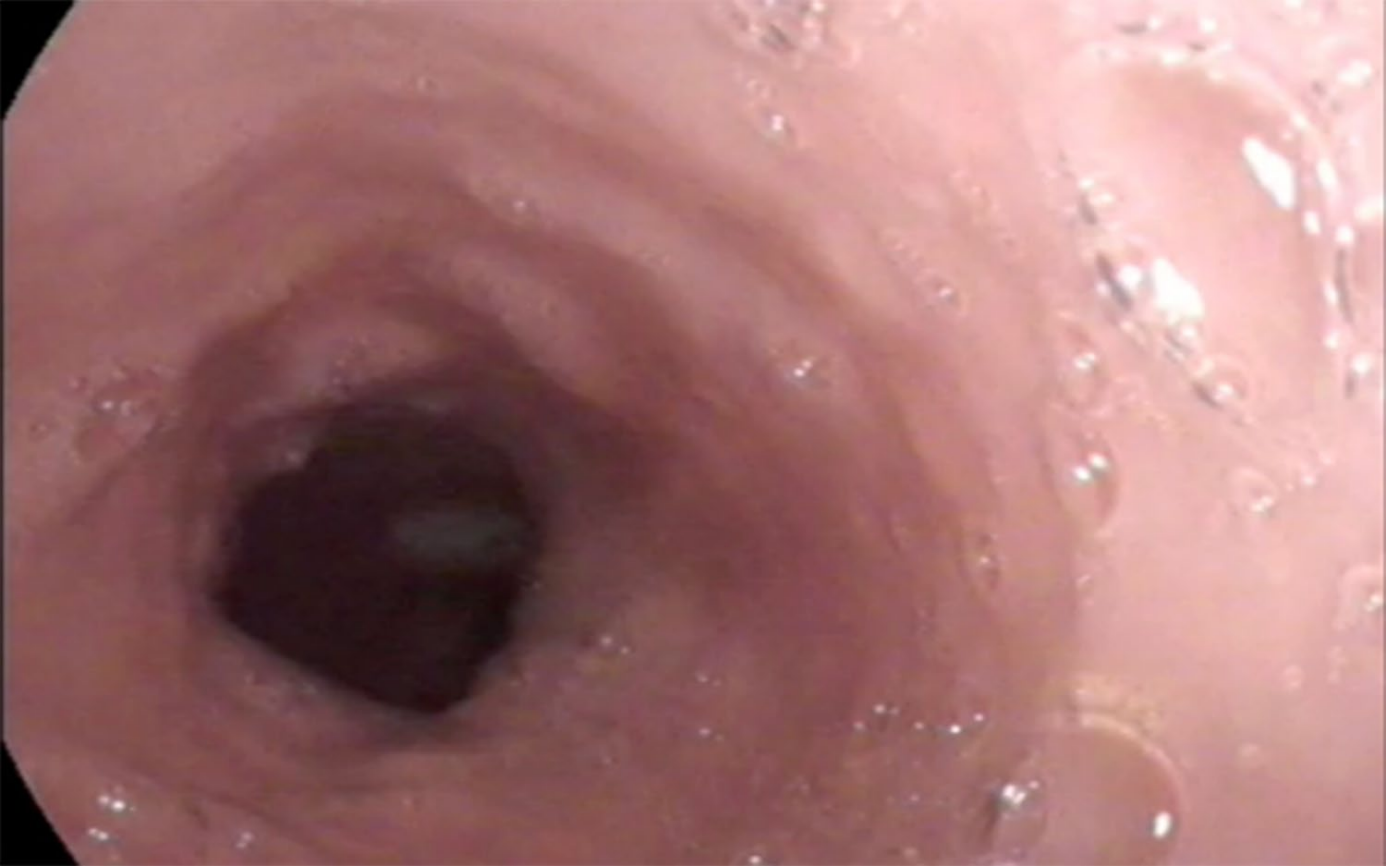
Transnasal oesophagoscopy showing gaseous distension of the oesophagus

**Fig. 2:**
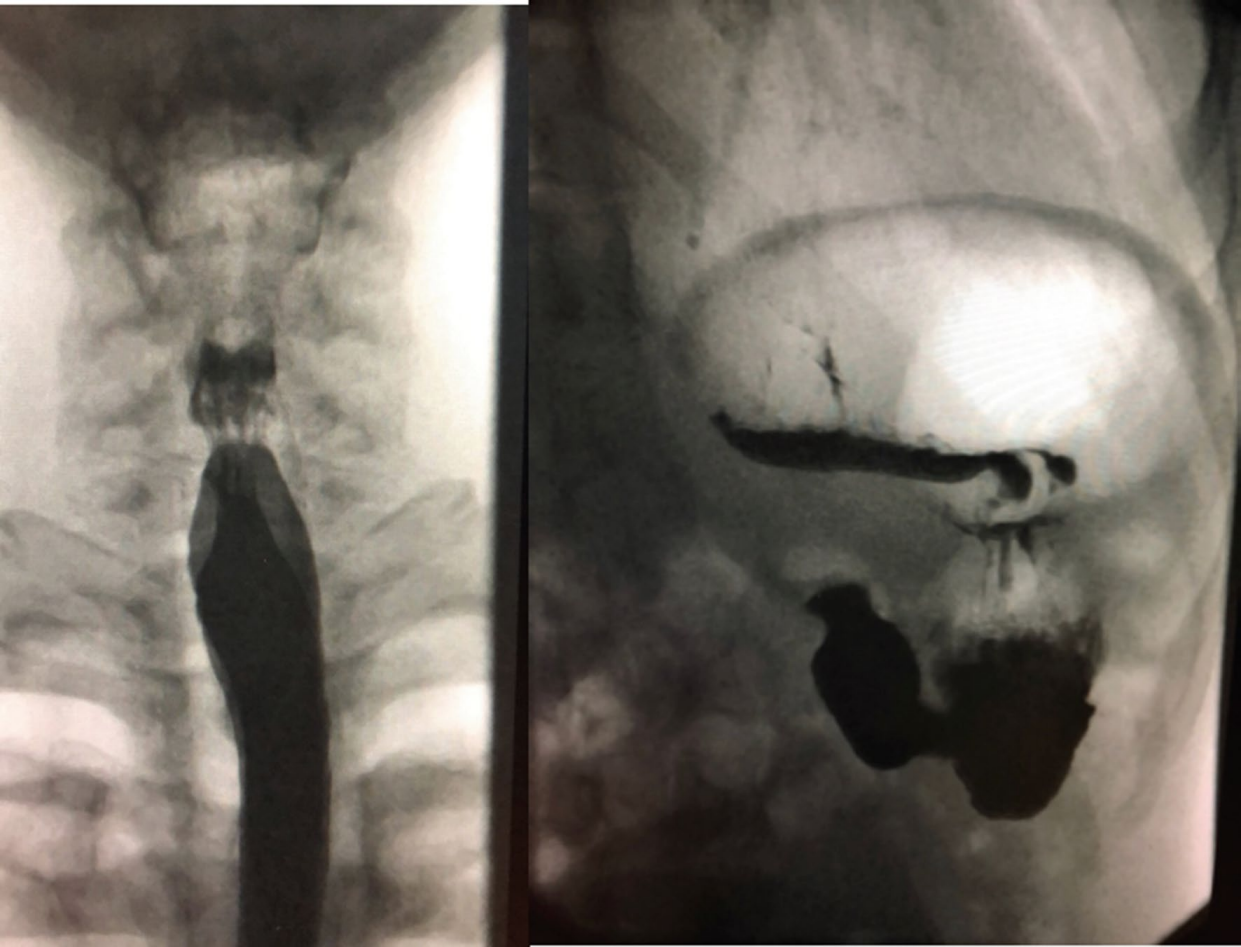
Barium swallow showing gas distended oesophagus and stomach

The diagnosis was made after a full clinical history and transnasal endoscopy of the pharynx and oesophagosocopy (TNO) in the outpatient set-up after a topical nasal spray anesthesia using 2.5 ml of 5% lidocaine with 5% phenylephrine in 0.5% water. The TNO showed a gaseous distended oesophagus (Fig. 1) which appeared to have reduced contractility in all cases. Some of the patients had barium swallow, which showed minor cricopharyngeal spasm and gaseous distention of the oesophagus and stomach (Fig. 2). Five patients had oesophagogastroduodenoscopy (OGD) and 2 had oesophageal pressure manometry elsewhere before presenting in my clinic and the results were essentially normal. Reflux Symptom Index was completed by 10 patients with average scores of 9 and EAT 10 score by five patients with average score of 2 and this is classified as within normal scores. In this retrospective cohort, the author did not routinely take these scores in all patients as the patients did not complain about significant reflux or dysphagia symptoms, so it was assumed that these scores are most likely irrelevant as it does not change the diagnosis or management of this condition.

**Fig. 3:**
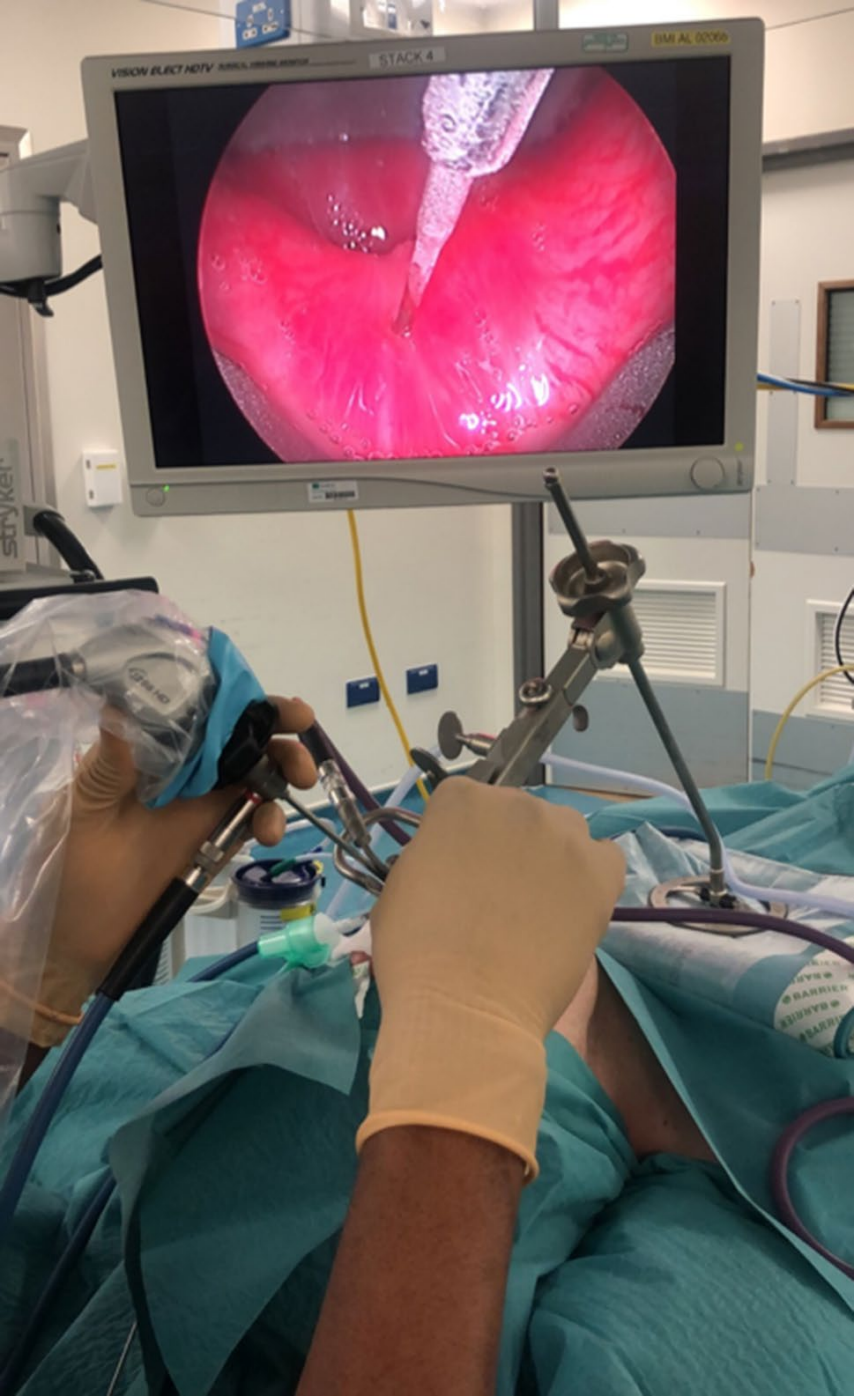
Suspension diverticuloscopy and video stack system and a 30 degrees rigid laryngeal telescope for visualising the pharynx

**Fig. 4:**
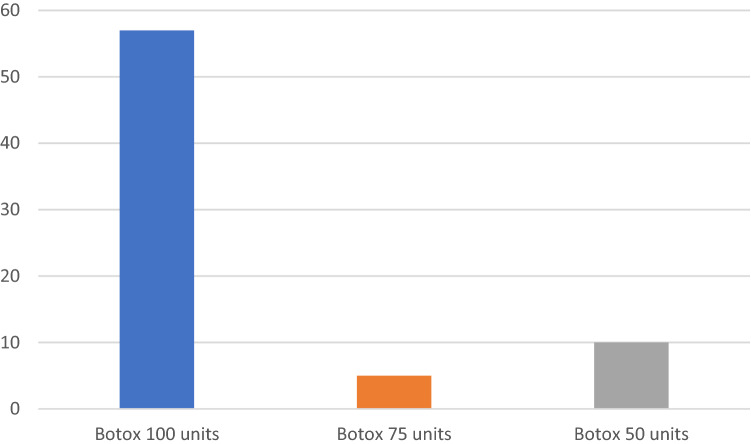
Number of patients injected with botox 100, 75 and 50 units

The surgery was performed under a general anaesthesia in supine position and the cricopharyngeal muscle was identified using a Suspension Weerda diverticuloscope. The procedure last about 30 min. Initially 50 units of botox diluted in 2 ml Normal saline was injected (1 ml in the posterior belly of the cricopharyngeus muscle and 0.5 ml each in the left and right posterior lateral aspects of the cricopharyngeus muscle) in the first 10 patients. And in the next five case series 75 units of botox diluted in 2 ml Normal saline was injected (1 ml in the posterior belly of the cricopharyngeus muscle and 0.5 ml each in the left and right posterior lateral aspects of the cricopharyngeus muscle) in the following five patients. Thereafter, 100 units of botox diluted in 2 ml Normal saline was injected (1 ml in the posterior belly of the cricopharyngeus muscle and 0.5 ml each in the left and right posterior lateral aspects of the cricopharyngeus muscle) for the remaining and this has been the authors practice to date (Figs. 3 and 4). As Bastian et al. [4] described in their case series, this treatment was more or less a therapeutic diagnosis as there was not sufficient existing literature about this condition. The reason for the gradual increment of the botox was because there has been delayed response of 2 weeks or more when 50 units or 75 units was injected. However, the response was quicker within 24–48 h when 100 units was injected.

## Results

A total of 72 patients were diagnosed and undergone surgery between November 2016 and December 2020. There were 50 male and 22 female patients. Their average age was 30 (range 18–68 years old). All patients were able to burp again within first 4 weeks of the injection. This persisted even after the Botox worn off beyond the 3 months in 96% of cases. The average follow-up was 24 months post injection with longest follow-up 48 months (range 1–48 months). None of the patients who had 100 units of botox at initial treatment required a repeat injection even after the presumed 3 months duration of action of botox. Of the three patients that had a recurrence within a month of Botox injection, two of them had 50 units and the 3rd patient had 75 units of the initial botox injection. Further Botox injection 100 units plus balloon dilatation of the cricopharyngeal sphincter using cook medical balloon size 20 mm × 2 (40 mm maximum) at 6 atmospheric pressure for 60 s was carried out under general anaesthesia in two of the patients that received 50 units of botox after 6 months of initial injection. Both patients had recurrence again in the fourth week after having a temporary relief.

No patients suffered any long-term complications. Most patients had mild and occasional regurgitation and effortful swallowing which lasted 1–2 weeks and gradually improved to complete normal by 4th week. No readmission required to hospital as a result of any complications. No cases of aspiration, stridor or hoarse voice were reported.

## Discussion

Although there has been a few single case series reported describing this condition [1–3], it was only recently that Bastian et al. [4] named this as retrograde cricopharyngeal dysfunction and presented the largest case series of 51 patients. Bastian et al. [4] also introduced for the first time the treatment of this condition using botulinum toxin A (botox) into the cricopharyngeal muscle with a long-term cure beyond the pharmaceutical period of the botox in over 90% of cases [5]. Furthermore, a single case report of a patient successfully treated with CO2 laser cricopharyngeal myotomy was reported by Bastian et al. [6] after recurrence following botox injection.

It is important that the treatment is replicated by other authors hence I reported the case series of 72 patients describing a similar condition. This is therefore the second largest case series aside from Bastian et al. [6] after searching the literature. In addition, I have reported the significance of using a larger dose of botox 100 units as standard dose. The reason for the increased dose of botox was because the initial 50 units of botox had a late response. All the patients injected with 100 units started to notice improvement that is, started burping within 48 h of injection all be it initial small burps but resume full burping by fourth week post injection. This effect lasted on average 24 months of the follow-up period.

The diagnosis of this condition is through history and clinical examination using a fiberoptic nasal endoscope and or trans nasal oesophagoscopy. Barium swallow, PH measurement and pressure manometry of the upper and lower oesophageal sphincters might be done to exclude a condition called achalasia, which is a narrowing of the gastro-oesophageal sphincter [7]. The laboratory and clinical findings in these patients might all be normal hence these patients are often told that the symptoms are psychological. For this reason, this is most likely dysfunctional condition as opposed to a physical or mechanical disorder as in achalasia or antegrade cricopharyngeal disorder due to pathologies like cricopharyngeal web or fibrosis that present with dysphagia. Some patients have already seen gastroenterologists and had oesophago-gastro-duodenoscopy (OGD), manometric and pH test and all were reported normal. Others were diagnosed as irritable bowel syndrome (IBS) or simple reflux but their symptoms did not improve after taking proton pump inhibitors and antacid or neither did they get any improvement after treatment for IBS. All patients said their social life was significantly affected and they avoid going out with friends and family due to fear of abdominal pain after eating. Two patients said they contemplated committing suicide as a result of these symptoms. Another patient had to were trousers with elastics waist as her waist line size changes throughout the day. One patient said she looks pregnant by the end of the day due to the excessive bloating of her abdomen. These findings are similar to that published by Bastian et al. [4]. There is a significant social and physical morbidity associated with this condition hence the need for clinician to recognise this whenever patients present with the above symptoms even in a normal laboratory finding.

None of the patients that had 100 units at initial injection reported recurrence. It is assumed that botox injection allows spontaneous expulsion of gas through the upper oesophageal sphincter and by the time the botox action wears off, the afferent–efferent feedback to the brain that initiate belching/burping get re-established.

Early recurrence was possibly due to failure of the afferent neural pathway to respond to the small dose of botox. However, it is unclear why even after a patent upper oesophageal sphincter following the maximum dilatation to 40 mm diameter, these patients still did not burp despite experiencing regurgitation.

## Conclusion

The author reports his experience in diagnosis and management of this rare condition of inability to burp and will like to suggest a simple term namely ‘Abelchia’ due to retrograde failure of the cricopharyngeal sphincter to relax spontaneously to release trapped oeosphageal gas. The author report that the relief of symptoms after botulinum toxin injection in the majority (96%) of cases diagnosed with this condition, outlasts the previously accepted duration of action of botulinum toxin injection (typically thought to be around 3 months).
